# Supplementary material for: Combined Targeted Omic and Functional Assays Identify Phospholipases A2 that Regulate Docking/Priming in Calcium-Triggered Exocytosis
Source: Cells. 2019 Apr 2;8(4):303. doi: 10.3390/cells8040303 (PMC6523306; doi:10.3390/cells8040303)
Supplement: Supplementary file 1 [file cells-08-00303-s001.pdf]

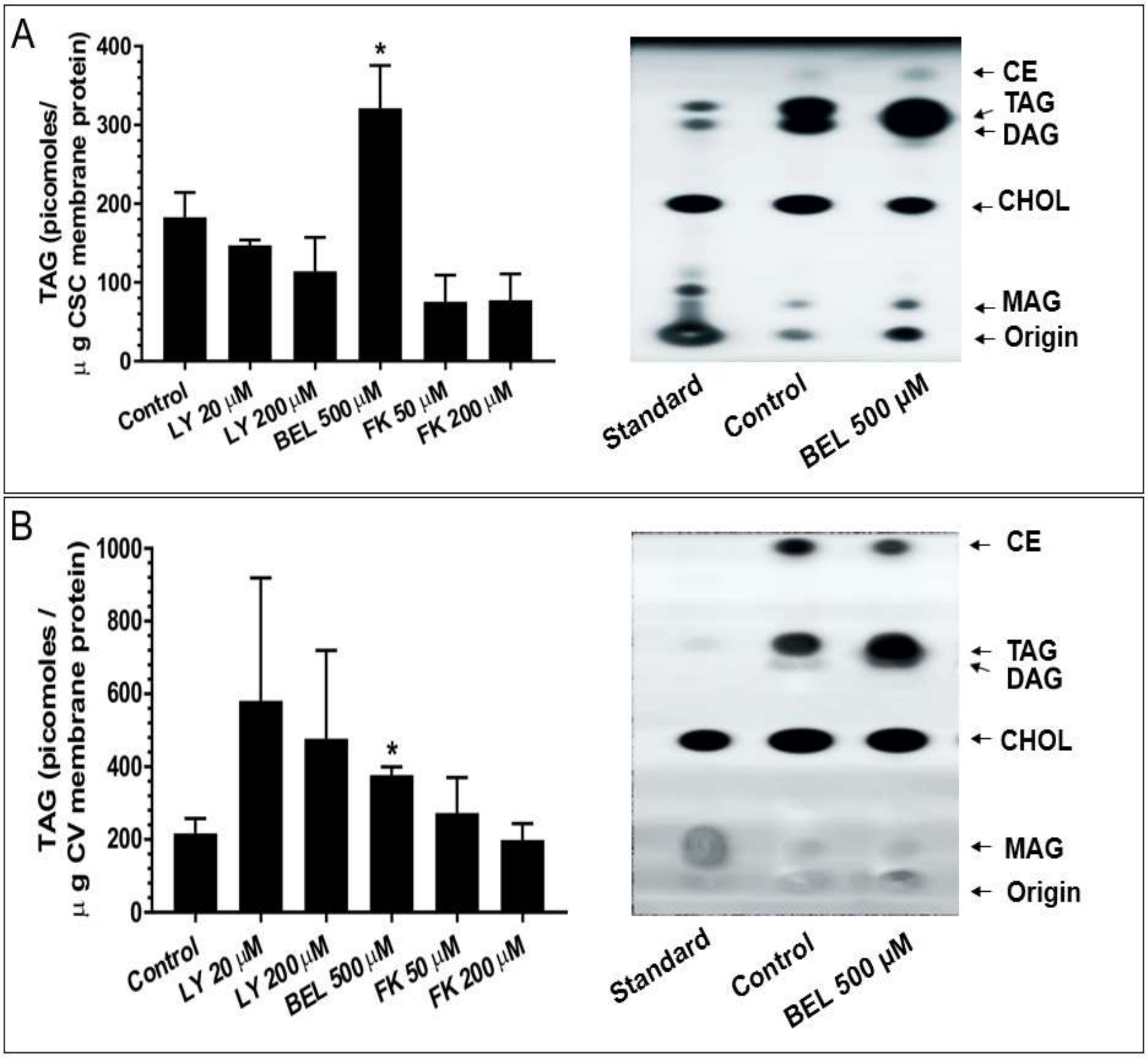

Figure S1: Endogenous TAG levels in (**A**) CSC, n = 3–9 and; (**B**) CV, n = 2–8 treated with the indicated doses of LY311727, BEL and FKGK-11 (representative chromatograms showing changes in neutral lipids). (p-value, * < 0.05, indicate difference relative to the control). A standard neutral lipid protocol was used to resolve the lipids [57].

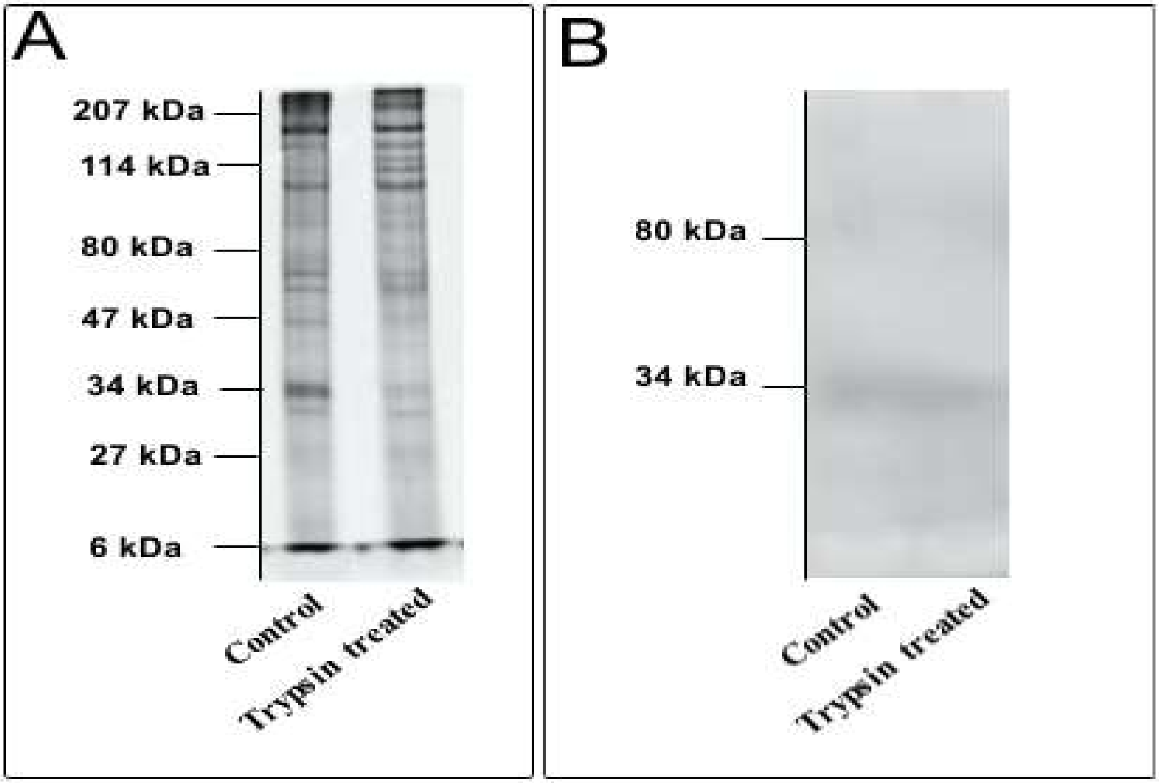

Figure S2: (**A**) SDS-PAGE of total membrane protein isolated from trypsin treated CV and a parallel untreated control stained with cCBB; (**B**) secondary antibody control western blot to identify non-specific protein binding (i.e. parallel control for western blot shown in Figure 6B). A total of 5μg CV membrane protein per lane was resolved.
